# The structure of a *Bacteroides thetaiotaomicron* carbohydrate-binding module provides new insight into the recognition of complex pectic polysaccharides by the human microbiome

**DOI:** 10.1016/j.yjsbx.2022.100084

**Published:** 2023-01-02

**Authors:** Filipa Trovão, Viviana G. Correia, Frederico M. Lourenço, Diana O. Ribeiro, Ana Luísa Carvalho, Angelina S. Palma, Benedita A. Pinheiro

**Affiliations:** a UCIBIO – Applied Molecular Biosciences Unit, Department of Chemistry, NOVA School of Science and Technology, Universidade NOVA de Lisboa, 2829-516 Caparica, Portugal; b Associate Laboratory i4HB - Institute for Health and Bioeconomy, NOVA School of Science and Technology, Universidade NOVA de Lisboa, 2829-516 Caparica, Portugal

**Keywords:** Human gut microbiota, Carbohydrates, Rhamnogalacturonan II, Carbohydrate binding module, *Bacteroides thetaiotaomicron*

## Abstract

The *Bacteroides thetaiotaomicron* has developed a consortium of enzymes capable of overcoming steric constraints and degrading, in a sequential manner, the complex rhamnogalacturonan II (RG-II) polysaccharide. BT0996 protein acts in the initial stages of the RG-II depolymerisation, where its two catalytic modules remove the terminal monosaccharides from RG-II side chains A and B. BT0996 is modular and has three putative carbohydrate-binding modules (CBMs) for which the roles in the RG-II degradation are unknown. Here, we present the characterisation of the module at the C-terminal domain, which we designated BT0996-C. The high-resolution structure obtained by X-ray crystallography reveals that the protein displays a typical β-sandwich fold with structural similarity to CBMs assigned to families 6 and 35. The distinctive features are: 1) the presence of several charged residues at the BT0996-C surface creating a large, broad positive lysine-rich patch that encompasses the putative binding site; and 2) the absence of the highly conserved binding-site signatures observed in CBMs from families 6 and 35, such as region A tryptophan and region C asparagine. These findings hint at a binding mode of BT0996-C not yet observed in its homologues. In line with this, carbohydrate microarrays and microscale thermophoresis show the ability of BT0996-C to bind α1-4-linked polygalacturonic acid, and that electrostatic interactions are essential for the recognition of the anionic polysaccharide. The results support the hypothesis that BT0996-C may have evolved to potentiate the action of BT0996 catalytic modules on the complex structure of RG-II by binding to the polygalacturonic acid backbone sequence.

## Highlights

• BT0996-C is a carbohydrate-binding module (CBM) from a modular RG-II-degrading enzyme.
• BT0996-C has a high content in positively charged residues, and absence of typical CBM35 and CBM6 binding signatures.
• Carbohydrate microarray and microscale thermophoresis reveals BT0996-C binding to α1-4-linked polygalacturonic acid (PGA).
• BT0996-C binding to PGA is driven by electrostatic interactions.

## Graphical abstract

**Figure:**
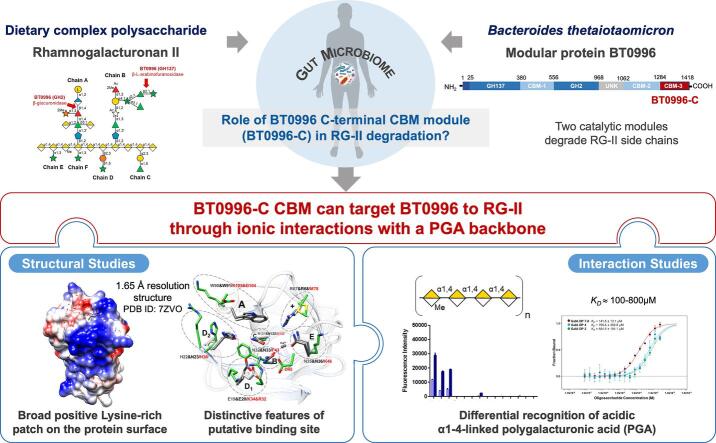

## Introduction

The human gastrointestinal tract houses a densely populated microbial community from almost all kingdoms of life (Ndeh and Gilbert, 2018). This complex and dynamic microbial community is known as the human gut microbiota and plays a crucial role in human health and nutrition (Wilson et al., 2020). The human gut microbiota is predominantly dominated by bacteria, in which a collection of beneficial symbionts is present (Martens et al., 2009).

One of the most common carbohydrate-active bacteria found in the human gut is the Gram-negative *Bacteroides thetaiotaomicron*, from the Bacteroidetes phylum (Desai et al., 2016). In *B. thetaiotaomicron* and other Bacteroidetes, carbohydrate utilisation is orchestrated by polysaccharide-utilisation loci (PULs) (Martens et al., 2011), which designate gene clusters encoding carbohydrate-degrading and -targeting systems. These systems include Carbohydrate-Active Enzymes (CAZymes) that are highly efficient to degrade complex dietary-derived carbohydrates, which otherwise would not be utilised by humans (Koropatkin et al., 2012, Martens et al., 2011). CAZymes often display a modular architecture where other domains frequently accompany the catalytic domain to provide additional carbohydrate recognition features. It has been also demonstrated that the number of enzymes in a given system directly correlates with the complexity of the targeted carbohydrate substrate (Grondin et al., 2017, Ndeh and Gilbert, 2018).

The pectic polysaccharide rhamnogalacturonan II (RG-II), which is present at the cell wall throughout the plant kingdom, is the most structurally complex dietary carbohydrate known, containing a wide diversity of monosaccharides and glycosidic linkages (Fig. S1) (O'Neill et al., 2004). RG-II is a branched polysaccharide with a backbone composed of at least eight α1,4-linked d-galacturonic acid (GalA) residues and four structurally well-defined oligosaccharide side chains (A-D) (Fig. S1) (Pellerin et al., 1996). RG-II is in its dimeric form in the primary cell wall structure, linked to α1,4-GalA homogalacturonans. Several studies predict the three-dimensional structure of the dimer to resemble two disks stacked together, where the chain A apiosyl residues from each monomer are covalently cross-linked by a borate diester (Kobayashi et al., 1996; Pérez et al., 2003).

In *B. thetaiotaomicron,* Ndeh and colleagues demonstrated that three different PULs are upregulated using RG-II as substrate (“RG-II degradome”) (Ndeh et al., 2017). These encode the necessary protein assembly to target and degrade RG-II efficiently. The degradation of RG-II is proposed to be hierarchical and to occur in the bacterial periplasm. The 23 enzymes that compose the RG-II degradome act in a sequential manner, each targeting a specific glycosidic linkage in the RG-II. The degradative process initiates with an *exo*-cleavage that targets first the RG-II side chains, then proceeds to remove the backbone and terminates with the final *exo*-cleavage of the remaining side chains. During this process, an enzyme that acts early in this hierarchical process is vital for the subsequent action of the cascade of enzymes (Ndeh and Gilbert, 2018, Ndeh et al., 2017).

**Fig. 1:**
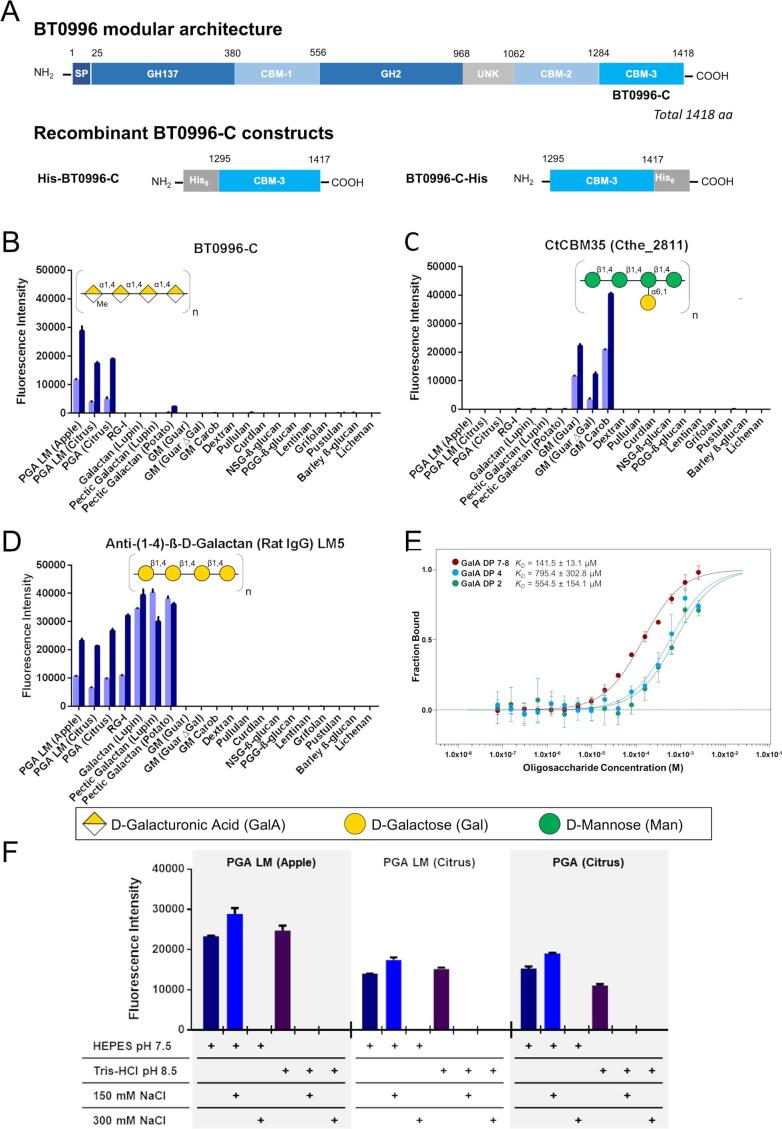
Exploring carbohydrate-binding by BT0996-C. A. Schematic representation of BT0996 native protein with delimitation of the different modules and the two protein constructs from BT0996-C CBM used in this work. (SP, Signal Peptide; GH, Glycosyl Hydrolase; CBM, Carbohydrate Binding Domain; UNK, Unknown function; His_6_, Histidine tag containing six histidine residues). Carbohydrate microarray analysis of (B) BT0996-C, (C) CBM35 (Cthe_2811) and (D) anti-(1–4)-β-d-Galactan (LM5) to plant-related polysaccharides. The binding scores are depicted as fluorescence intensities elicited with 30 and 150 pg (by weight) polysaccharide per spot (light blue and dark blue, respectively). The major oligosaccharide sequence domains present in the bound polysaccharides are represented using the updated symbol nomenclature for glycans (SNFG). PGA, Polygalacturonic Acid; LM, Low Methoxyl; RG-I, Rhamnogalacturonan I; GM, Galactomannan; NSG, Neutral Soluble Glucan; PGG, Poly-(1,6)-d-glucopyranosyl-(1,3)-d-glucopyranose. E. Initial fluorescence analysis of BT0996-C interaction with PGA derived oligosaccharides with the degree of polymerisation (DP) 2–8 α1,4-galacturonic acid units. To obtain the K_D_ values, dose–response curves were fitted to a one-site binding model. Error bars indicate the standard deviation of quadruplicate (n = 4). F. Carbohydrate microarray analysis of BT0996-C at different ionic strengths to PGA polysaccharides.

In one of those PULs (PUL 92), a modular enzyme (BT0996) with bi-functional activity was identified as being involved in the initial *exo*-cleavage stage of RG-II deconstruction (Ndeh et al., 2017). BT0996 comprises two catalytic domains and three putative carbohydrate-binding modules (CBM-1–3, Fig. 1A). The BT0996 β-d-glucuronidase (GH2) and β-l-arabinofuranosidase (GH137) target RG-II chains A and B, respectively, in a coordinated manner by cleaving monosaccharide decorations from the terminal regions of each chain. The role of BT0996 is in the early stages of the degradation process, being the first enzyme to cleave the RG-II side-chain B and the second on the RG-II side-chain A. The BT0996 GH137 module does not need an upstream enzyme for removing the RG-II side-chain B terminal arabinose but BT0996 GH2 needs the early removal of l-galactose from side-chain A by BT1010 before cleaving of the glucuronic residue from this same side-chain (Fig. S1). The removal of the l-galactose-d-glucuronic acid disaccharide causes destabilisation of the borate di-ester cross-links with d-Apif (present in side-chain A) and leads to the disassembly of the RG-II dimer. After this event, steric constraints are released, and other enzymes can continue with the RG-II degradation (Ndeh et al., 2017).

Although abundant information concerning the action of the two catalytic modules of BT0996 exists, not much is known about the putative CBM modules and their roles in the RG-II degradation. These non-catalytic domains are frequently found attached to enzymes and operate as substrate-binding modules. CBMs are reported to mediate the specific targeting and recognition of the carbohydrate substrate, enhancing enzyme proximity and substrate degradation efficiency (Abbott and van Bueren, 2014, Boraston et al., 2004, Lombard et al., 2014, Sidar et al., 2020). Here, we report the characterisation of the CBM-3 C-terminal domain of BT0996 protein, here designated BT0996-C, using X-ray crystallography combined with carbohydrate microarrays and microscale thermophoresis (MST). The high-resolution crystal structure of BT0996-C shows distinctive features in the putative ligand-binding site, impacting its carbohydrate-binding specificity. The affinity to polygalacturonic acid (PGA) discloses as a possible function the approaching of BT0996 to anionic pectic substrates, such as RG-II.

## Results and discussion

### BT0996-C interacts with the α1,4-linked polygalacturonic acid backbone of pectic polysaccharides

To select and delimitate non-catalytic domains with potential carbohydrate binding, an InterPro and BlastP search of the BT0996 protein was performed, revealing the presence of three putative carbohydrate binding modules – CBM-1, CBM-2 and CBM-3 (Fig. 1A). The CBM-1 module has sequence similarity to galactose binding domains, while CBM-2 has sequence similarity to malectin and is assigned to the CBM family 57 in the CAZY database. The CBM-3 (BT0996-C, 1284–1418) is assigned as belonging to the CBM6- and CBM35-like superfamily in the Conserved Domain Database (CDD). Recombinant expression of the three modules was attempted, but CBM-1 and CBM-2 failed soluble expression and were not further characterized. The delimitated BT0996-C gene fragment (1295–1417) was cloned in the NZYTech pHTP1 vector with an *N*-terminal His-tag and further re-cloned with a C-terminal His-tag (Fig. 1A). Both constructs were recombinantly expressed in *Escherichia coli*, purified and used in the biochemical studies and crystallization trials. Different protein buffers were used for purification and storage accordingly (Table 1).

**Table 1:** Purification and Storage Buffers of BT0996-C.

**Protein constructs:**	**His**-BT0996-C	BT0996-C**-His**
**Buffers for crystallisation purposes**	Purification Buffers (Start and Elution)*	
	20 mM Tris-HCl, pH 8.5	50 mM HEPES, pH 7.5
	Storage Buffers**	
	20 mM Tris-HCl, 5 mM CaCl_2_, pH 8.5	100 mM Sodium Citrate, 25 mM Glucose, 1 mM CaCl_2_, pH 5.5

**Buffers for Interaction Experiments**	Purification Buffers (Start and Elution)*	
	50 mM HEPES, pH 7.5	
	Storage Buffer**	
	50 mM HEPES, 5 mM CaCl_2_, pH 7.5	

*All purification buffers contained 1 M NaCl, 5 mM CaCl_2_ and 5 mM β-mercaptoethanol. Start buffers contained 10 mM Imidazole and Elution buffers contained 300 mM Imidazole.

**All storage buffers contained 150 mM NaCl and 5 mM DTT.

To screen BT0996-C for carbohydrate binding, we performed screening analysis using plant-related polysaccharide samples isolated from different sources in a microarray setup (Table S1). The polysaccharide microarray was also analysed with characterised proteins, which showed binding profiles according to their reported carbohydrate-binding properties and validated the constructed microarray (Fig. S2 and Table S2).

BT0996-C showed exclusive binding to homogalacturonan polysaccharides with an α1,4-linked galacturonic acid (GalA) backbone chain (polygalacturonic acid, abbreviated PGA in Fig. 1B and Table S3). No interaction was observed with rhamnogalacturonan-I (RG-I), a branched polysaccharide constituted by a backbone of repeating α1,4-linked disaccharide units of l-rhamnose and GalA. The analysis with antibody LM5 showed immobilization of this polysaccharide on the microarray, binding to the polysaccharide through recognition of its β1,4-d-Galactan side chain (Fig. 1D). The binding profile of BT0996-C contrasted with that obtained with a homologous β-mannan-specific CBM35 from *Clostridium thermocellum* (CtCBM35_Cthe_2811_) (Ghosh, et al, 2014), which showed the predicted binding to galactomannans (Fig. 1C).

The quantitative analysis of the interaction of BT0996-C with homogalacturonan polysaccharides and oligosaccharides was initially investigated using isothermal titration calorimetry (ITC). The attempt was unsuccessful due to the low heat release (data not shown). This suggests that there is a significant entropic contribution, enough for cancelling the enthalpic contribution and the heats not being observable by ITC. To circumvent this problem, we used microscale thermophoresis (MST) which, contrary to ITC, does not rely on heat measurements for quantifying biomolecular interactions. Here, we labelled BT0996-C with a fluorescent dye and used the fluorescence changes observed as a direct result of the non-fluorescent ligand increasing concentrations to determine the dissociation constants (Jerabek-Willemsen et al., 2011). The comparison of the binding curves shows that BT0996-C has a preference for longer oligosaccharides (Fig. 1E, Table 2), revealing a chain-length dependency of the interaction.

**Table 2:** The affinity of the interaction between BT0996-C-His and the different homogalacturonan oligosaccharides was obtained through initial fluorescence analysis of microscale thermophoresis experiments, which were performed in quadruplicate (n = 4). The fitting quality is given by the standard error of the regression and reduced chi-square (χ^2^) parameters.

Ligand	*K*_D_ ± error (μM)	[Ligand] (mM)	[Protein] (nM)	n replicates	Std. Error of Regression	Reduced χ^2^	S/N ratio
**Galacturonate-7**–**8**	141.5 ± 13.1	2.5	66	4	7.60	5.60	40.02
**Galacturonate-4**	795.4 ± 302.8	2.5	66	4	4.47	0.54	16.31
**Galacturonate-2**	554.5 ± 154.1	2.5	66	4	6.07	1.23	18.95

To confirm the role of ionic interactions in mediating the interaction of BT0996-C with a backbone sequence of α1,4-linked GalA, we analysed the binding of BT0996-C to pectin polysaccharides using two buffers with different ionic strength (HEPES pH 7.5 and Tris-HCl pH 8.5) and with increasing concentrations of salt concentrations (NaCl 0, 150 and 300 mM). BT0996-C showed binding to PGA samples at low ionic strength, while an abrogation of interaction was observed at higher salt content (Fig. 1F and Table S4). Tris-HCl is the buffer with a higher conductivity value (Reijenga et al., 1996) used in this experiment, and the addition of 150 mM NaCl was sufficient to impair the BT0996-C interaction with the PGA samples. This experiment showed that buffers with high ionic strengths could neutralise the electrostatic interactions of BT0996-C, providing evidence for their ionic nature. Thus, we confirmed that the recognition of the α1,4-galacturonic acid backbone has a significant contribution from the electrostatic interactions established between the basic side chains of the lysine and arginine residues and the acidic groups of the galacturonic residues.

Some carbohydrate-binding proteins specifically recognise complex charged carbohydrates through electrostatic interactions (Gandhi and Mancera, 2008). This seems to be the case of BT0996-C, which recognises PGA but no other tested polysaccharide also rich in galacturonic acid such as RG-I (Table S1).

### Crystal 3D structure of BT0996-C

To understand the molecular determinants of the interaction of BT0996-C with PGA, the two constructs were both submitted to crystallisation trials, generating both good diffracting crystals. The crystal structure of BT0996-C with the *N*-terminal His-tag was determined at 1.65 Å resolution by molecular replacement using, as phasing model, the preliminary 2.3 Å resolution structure of the C-terminal His-tag construct, solved by Se-SAD (Table S5). The final refined high-resolution structure contained one molecule of BT0996-C in the asymmetric unit, consisting of 122 amino acid residues of the wild-type protein and an additional nine residues of the *N*-terminal tag.

**Fig. 2:**
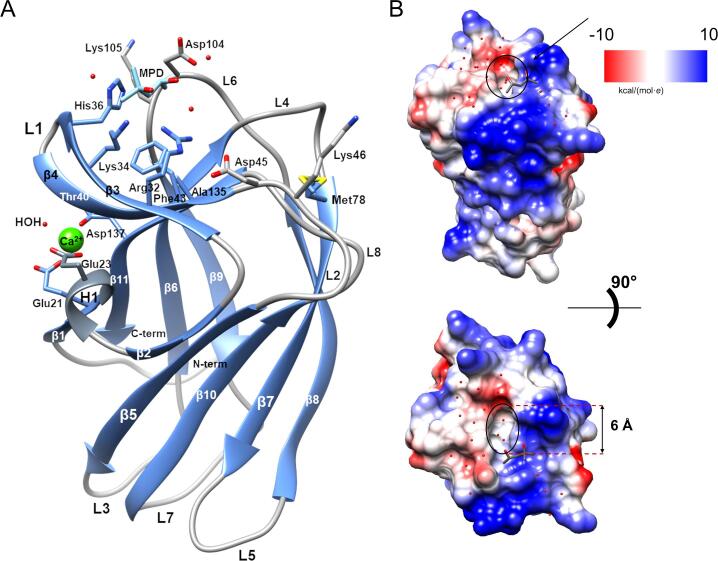
The 3D structure of BT0996-C CBM module from *Bacteroides thetaiotaomicron VPI-5482*. A. Ribbon representation of BT0996-C displaying the side-chains from relevant residues of the putative binding site and Calcium coordination. Strands are shown in blue, loops in grey and Helix H1 in dim grey. The Calcium (II) ion is represented by a green sphere and the MPD molecule in cyan. B. Representation of the electrostatic surface charges distribution of BT0996-C from two perspectives. Negatively charged atoms are coloured red, and positively charged atoms are coloured blue. A neutral pocket, marked by a line circle and with a diameter of approximately 6 Å, is observed in the putative binding site. The structural representation and surface electrostatic potential calculations were performed with UCSF Chimera (Pettersen et al., 2004).

BT0996-C displays a distorted *β*-sandwich (Fig. 2A), composed of two *β*-sheets connected by loops. *β*-Sheet A includes *β*-strands 1, 3, 4, 11, 6 and 9, whereas *β*-sheet B comprises *β*-strands 2, 5, 10, 7 and 8. The structural calcium ion, highly conserved in many lectins and CBM families (Correia et al., 2010, Sainz-Polo et al., 2014), is positioned near β-1, β-11 strands and H1 helix. This metal ion is coordinated by Glu21 Oε1, Glu23 Oε1 and Oε2, Asp137 Oδ1, the backbone carbonyls of Asp137, Thr40, and one water molecule, in an octahedral geometry confirmed by the Check My Metal server (Zheng et al., 2014, Fig. S3A).

A striking feature of BT0996-C is its high content in positively charged residues (15 Lys, 2 His and 4 Arg), representing almost 20 % of the protein composition, resulting in a theoretical isoelectric point (pI) of 9.43. This feature confers a prominent broad positive patch to the electrostatic surface of the BT0996-C protein (Fig. 2B), which includes both *β*-sheets and the loops that connect them. Another unusual trait is the absence of a cleft or groove with exposed aromatic residues which is a hallmark of CBM modules. In BT0996-C structure we could only observe a negatively charged cleft with a tyrosine in its proximity.

### The putative binding site of BT0996-C shows distinctive features related to its carbohydrate-binding specificity

To target the putative binding site of BT0996-C, the protein was incubated with different monosaccharides present in the RG-II structure (Table S6) and co-crystallisation setups were prepared. X-ray diffraction data from crystals of the incubated protein produced no electron density that could be assigned to any bound monosaccharide. Instead, in the 3D structure here reported, a 2-methyl-2,4-pentanediol (MPD) molecule present in the crystallisation solution could be modelled, establishing CH–π interactions to His36 and water-mediated hydrogen bonds to Gly133 O and Thr73 Oγ1 from *β*-sheets 3 and 4, near the loop region (Fig. S3B). This *β*3-L1_loop_-*β*4 region (Lys33-Thr44) was highly disordered in the BT0996-C Se-Met derivative structure, where electron density from residues 11 to 39 was missing due to disorder (data not shown). This stretch of disordered residues included the calcium coordination region. The presence of the MPD molecule stabilised this region and consequently the calcium ion coordination region.

**Fig. 3:**
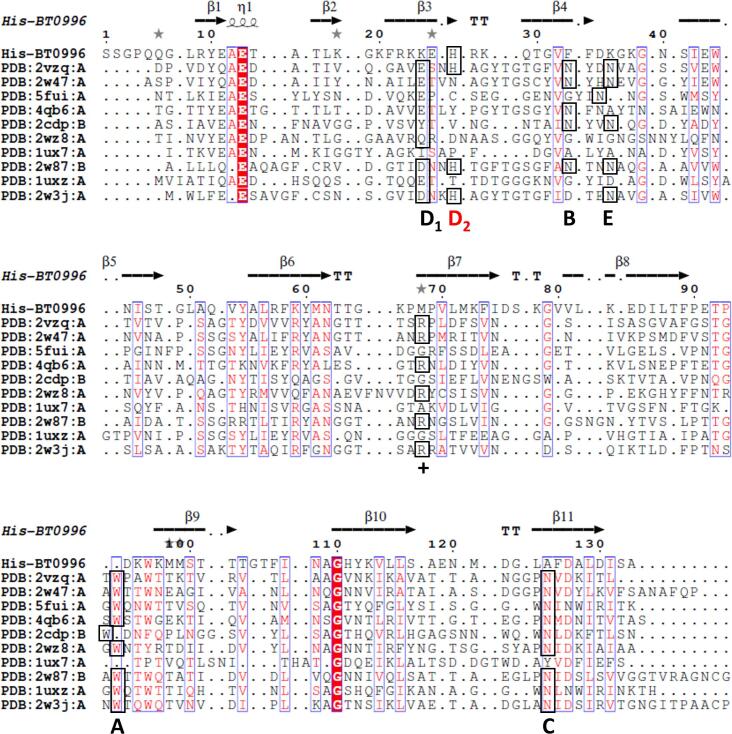
Multiple structure-informed primary sequence alignment of BT0996-C with homologue CBMs of known three-dimensional structures: 2vzq, Chi-CBM35 of the *exo*-β-d-glucosaminidase from *A. orientalis;* 2w47*,* Rhe-CBM35 of rhamnogalacturonan acetyl esterase from *C. thermocellum*; 5fui, ZgLamC-CBM6 of the laminarinase from *Zobellia galactanivorans*; 4qb6, Xyn30D-CBM35 of the glucuronoxylanase Xyn30D from *Paenibacillus barcinonensis*; 2cdp, Aga16B-CBM6-2 of the β-agarase from *Saccharophagus degradans 2*–*40*; 2wz8, CBM35 binding d-galactose from *C. thermocellum*; 1ux7, CBM36 domain of the *Paenibacillus polymyxa* xylanase; 2w87, Xyl-CBM35 of the xylanase CjXyn10B from *Cellvibrio japonicus*; 1uxz, *Cm*CBM6-2 of endoglucanase 5A from *Cellvibrio mixtus*; and 2w3j, Pel-CBM35 a PL10 pectate lyase (Pel10) from an environmental isolate. Relevant amino acid residues present in the binding site (Regions A-E and basic path+) are labelled below the sequences. Conserved amino acids are highlighted in boxes. In red, Region D_2_, is the only residue conserved in BT0996-C. PDBeFold was used to search the homologue CBM structures and structural alignment prepared using the ESPript 3 online tool.

The observation of an MPD molecule in this region suggests a putative binding site, as the binding sites of BT0996-C homologues are also located in the extended loops that connect the two β-sheets. A PDBeFold search for structurally similar proteins resulted in several CBMs belonging to family 35 with binding specificity for uronic acids, but also CBMs from family 6 and one from family 36 (Correia et al., 2010, Montanier et al., 2009, Sainz-Polo et al., 2014) (Table S7, Fig. 3). The CBMs with closest structural similarity to BT0996-C are two CBM35 (Montanier et al., 2009), followed by a CBM6 (Jam et al., 2016), sharing sequence identities of 21 %, 22 % and 17 %, respectively.

**Fig. 4:**
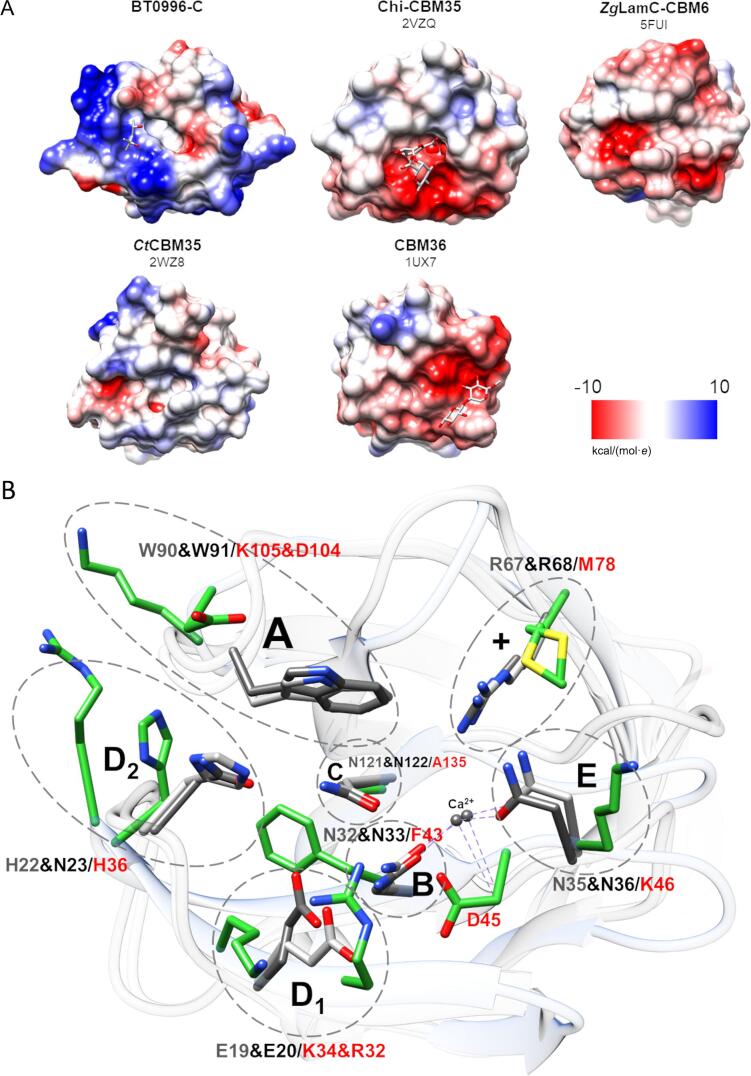
Exposing BT0996-C differences from structurally similar CBMs. A. Representation of the electrostatic surface charges distribution of the (putative) binding sites from BT0996-C and representative homologue CBMs. Negative charges are coloured red, and positive charges are coloured blue. B. Secondary structure matching superposition of BT0996-C CBM and the two family 35 CBMs of higher structural similarity. Amino acid residues involved in binding site formation are displayed as sticks and metal ions as grey spheres. Carbon atoms are shown in green for BT0996-C, light grey for 2VZQ (Chi-CBM35), and dim grey for 2W47 (Rhe-CBM35). Functional regions are circled with dashed lines and labelled. The surface calculations and structural illustration were performed with UCSF Chimera (Pettersen et al., 2004).

A close inspection of common carbohydrate-interacting aromatic residues like tryptophan and tyrosine present on CBMs and other carbohydrate-binding proteins and known to mediate the recognition with sugar residues through π-CH stacking interactions, showed that these are absent from BT0996-C’s putative binding site. Instead, His36 and Phe43 were found, which are more electron-poor aromatic amino acids and then less prone to make CH–π interactions with sugar residues (Hudson et al., 2015). Notably, the surface shape and charge of the BT0996-C putative binding site distinguishes it from homologue CBMs. When comparing ten structurally similar CBMs, it was observed that BT0996-C exhibits a more positively charged binding site (Fig. 4A). The positively charged amino acid residues create protrusions in the surface shape that border an unusual small hydrophobic pocket comprising Ala135 and Phe43 in the centre.

The diverse specificities observed for CBM members from families 35 and 6 seem related to the structural diversity in the surface shape of their binding sites (Abbott and van Bueren, 2014). For CBM35 and CBM6 families five key regions (A-E) were assigned in the binding site, which are known to be essential for ligand binding and specificity (Abbott and van Bueren, 2014, Correia et al., 2010). These key regions were inspected in BT0996-C's crystal structure and compared with structurally similar proteins solved with the respective ligands (Fig. 4B). Regions A and C are the most conserved in both CBM35 and CBM6. Region A is usually dominated by a Tryptophan residue, responsible for the π-CH stacking interaction with the sugar residue, while the invariant region C shows an Asparagine residue at the base of the binding site that, in known complex structures, makes critical hydrogen bonds with hydroxyl groups of the sugar residue. The BT0996-C’s 3D structure shows residues with dissimilar chemistries in both regions. The aromatic residue is absent in region A, replaced by Asp104 and Lys105, while Ala135 is found in region C. Region B is more diverse depending on the subfamily and in uronate-interacting CBM35 modules, it is associated with a metal coordination site (asparagine). BT0996-C does not have this metal coordination site since, instead of asparagine in this region, BT0996-C has a Phenylalanine residue. This indicates that if indeed the interaction with the ligand occurs in this region, it is probably not mediated by a calcium ion. Region D subdivides into two sub-regions defined as D_1_ and D_2_ (Abbott and van Bueren, 2014, Correia et al., 2010). BT0996-C seems to conserve a higher similarity in this region when compared with other described members of the CBM35 family. In known complex structures, region D_1_ is usually dominated by polar residues that make the binding site more pocket-like, while in BT0996-C, Lys34 and Arg35 are found. Region D_2_ is characteristic of uronate-specific CBM35 and usually has a conserved Histidine residue that interacts with the uronic residue through hydrogen bonds. This residue (His36) is also found in region D_2_ of BT0996-C. Finally, Region E also displays a high degree of variation and contributes to ligand specificity among CBM35 and CBM6 family members. In CBM35, an Asparagine is frequently found in this region, near a basic path (Arginine, +). Both features are absent in the BT0996-C structure. Instead, a Lys46 can be found in region E near Met78.

### BT0996-C is a novel and unique CBM that recognises PGA

BT0996-C has structural similarity to CBM modules from families 35 and 6, characterised for having a variety of specificities that may not entirely match to their adjacent catalytic modules – a direct consequence of the variety of their binding-sites topologies (Montanier et al., 2009, Correia et al., 2010; Tunnicliffe et al. 2005; Fujimoto et al., 2017). Despite the similarity, the BT0996-C structure holds a novel and extended carbohydrate-binding site with a high percentage of positively charged residues, with a high content in Lysine residues, which explains the differential recognition of α1,4-linked polygalacturonic acid. A further refinement of the carbohydrate-binding specificity of this CBM is needed using purified RG-II oligosaccharide fragments to conclude about differential and specific recognition of RG-II among pectic polysaccharides. In addition to being present in the RG-II backbone, homogalacturonan chains are associated with RG-II in the plant cell wall. As BT0996 acts in the early stage of RG-II degradation when it is in its most recalcitrant form, BT0996-C ability to recognise the highly abundant α1,4-linked polygalacturonic acid in the plant cell wall might be important to ensure that BT0996 catalytic modules reach their substrates in the RG-II complex structure, initiating the degradation process.

## Methods

### BT0996-C cloning, expression and purification

The *BT0996-C* gene was amplified from *Bacteroides thetaiotaomicron* genome and cloned into the pHTP1-A53 vector by NZYTech company established protocols (Sequeira et al., 2017). The HTP construct (His-BT0996-C) has an *N*-terminal His-tag (with six histidine residues) and antibiotic resistance to kanamycin. A second construct (BT0996-C-His) was designed to improve the probability of obtaining protein crystals. To generate the second construct, the recombinant DNA from the HTP construct was re-cloned into a *Nco*I- and *Xho*I-restricted pET28 vector with a C-terminal His-tag. The functional and structural studies of CBM BT0996-C were performed using these two protein constructs in different steps. The oligonucleotides used for cloning and re-cloning of BT0996-C are listed in Supplementary Table S8.

#### His-BT0996-C and selenomethionine BT0996-C-His expression

His-BT0996-C was expressed in Tuner (DE3) *E. coli* strain. Cell culture was incubated in Luria-Bertani (LB) medium with 50 µg/mL kanamycin at 37 °C, with shaking. The induction was carried out with 0.2 mM isopropyl-β-d-thiogalactopyranoside (IPTG) when cells were at mid-exponential growth phase (OD_600nm_ = 0.6), and cell cultures were incubated at 37 °C, for 3 h. Then, cells were harvested by centrifugation at 6000×*g* for 15 min (4 °C). For selenomethionine-labelled BT0996-C-His production, SelenoMet™ Medium from Molecular Dimensions Limited was used, following the instructions provided by the supplier. BT0996-C-His construct was transformed in methionine auxotroph *E. coli* B834 (DE3) strain. Expression of BT0966-C-His was induced by the addition of 1 mM IPTG at OD_600nm_ = 0.6, and cells were incubated at 37 °C for 5 h. Cells were collected by centrifugation.

#### Protein purification

The purification protocol for both protein constructs was performed by immobilised-metal affinity chromatography (IMAC) using a HisTrap™ column (5 mL) with Ni^2+^, coupled to the chromatograph ÄKTA START (GE Healthcare). The cells pellet was resuspended in their respective Start Buffer (Table 1) containing protease inhibitors, DNaseI (5 µg/mL), 5 mM MgCl_2_ and Lysozyme (300 µg/mL). Cells were disrupted by sonication (UP100H, Hielscher Ultrasonics) and the crude extract centrifuged at 13,000×*g* for 30 min (Eppendorf, Centrifuge 5804K). The supernatant was loaded into the column, and the first wash with 50 mM imidazole was applied. Protein was eluted using an imidazole gradient from 50 to 500 mM. All fractions were analysed by SDS-PAGE and pooled together. Proteins were buffer-exchanged to an appropriate storage buffer (Table 1) with no imidazole, using HiTrap Desalting columns with Sephadex G-25 resin (GE Healthcare), coupled to the ÄKTA START. Purified proteins were concentrated by ultrafiltration using Vivaspin® Turbo 15 (Sartorius) with a 3 kDa cut-off membrane.

### Protein storage buffer optimisation through Thermal Shift assay (TSA)

To improve protein stability and sample homogeneity, a storage buffer optimisation was performed using Thermal Shift Assay (Boivin et al., 2013). For this, two screens were performed, first a screen with different buffers at different pH and NaCl concentrations, and then the RUBIC additive screen (Molecular Dimensions), composed of a wide range of small molecules. To perform TSA, 96-well plates were used (MicroAmp® Fast 96-well Reaction Plate (0.1 mL) from Applied Biosystems). A reaction mixture of 20 µL was prepared in each well: 10 µL of the buffer/additive solution to be screened, 2 µL at 40 µM of protein, 3 µL of dye 8×, prepared in milli-Q-H_2_O (Thermal Shift Dye Kit™, Applied Biosystems) and 5 µL of milli-Q-H_2_O/Buffer 4× was added and mixed. The plate was sealed and centrifuged (1 min, 200 g, 4 °C). TSA was performed in a StepOnePlus Real-Time PCR system (Applied Biosystems) with ROX (575/602 nm, absorption/emission) filters. The temperature was scanned from 25 to 95 °C, at 1 °C/min. Data was exported, and each melting curve and respective melting temperatures (Tm) were analysed.

### Protein crystallisation assays

Crystallisation experiments were conducted using an automated crystallisation nanodrop robot (Oryx8, Douglas Instruments) on 96-well crystallisation plates, where 0.67 µL of protein was mixed with 0.33 µL of reservoir solution. Commercial crystallisation screens were used: JCSG-plus, Structure 1 and 2, and Morpheus at two different temperatures (4 °C and 20 °C). For selenomethionine BT0996-C-His, crystals grew in a drop with 16.6 mg/mL of protein in Sodium Citrate buffer against a reservoir containing 50 µL of 40 % (w/v) polyethylene glycol 300, 0.1 M sodium cacodylate pH 6.5 and 0.2 M calcium acetate, at 20 °C. To obtain His-BT0996-C structure in complex with a monosaccharide present in RG-II, co-crystallisation trials were tried with a mix of monosaccharides (MonoS) (Table S6). For this, 5 mM of each MonoS was incubated overnight at 4 °C with 16 mg/ml (1 mM) of protein. The best crystals appeared in the Morpheus screen at 4 °C, in a drop containing 8 mg/mL of protein with 2.5 mM of MonoS mix. The reservoir contained 50 µL of 0.1 M Amino acids mix (dl-Glu, Ala, Gly, Lys, Ser), 0.1 M Buffer system 3 (Tris, Bicine pH 8.5) and 37.5 % Precipitant Mix 4 (25 % MPD, 25 % PEG 1000, 25 % PEG 3350). Both protein crystals were harvested after 2 months and flash-frozen in liquid nitrogen using Paratone-N oil as cryoprotectant.

### X-ray diffraction data collection, structure solution and refinement

#### SeMet BT0996-C-His

For SeMet BT0996-C-His crystals, one Single-wavelength Anomalous Diffraction (SAD) data set was collected, to a maximum resolution of 2.43 Å on beamline ID30B at the European Synchrotron Radiation Facility (Grenoble, France), using 0.97937 Å wavelength radiation. Experimental values of the anomalous-scattering factors, f′ = −7.75e and f″ = 5.76e, were derived from the X-ray fluorescence scan using Chooch (Evans and Pettifer, 2001). The BT0996-C-His crystal indexed in space group *P*4_1_22 with cell constants *a* = 49.14 Å, *b* = 49.14 Å, and *c* = 282.89 Å. Matthews Coefficient calculations suggested the presence of 2 copies of BT0996-C-His in the asymmetric unit, with V_M_ = 2.84 Å^3^Da^−1^ and an approximate solvent content of 57 %. Se-phasing was successfully achieved using AutoSol (Terwilliger, 2004), implemented in Phenix (Adams et al., 2010). The best phasing solution (Bayes-CC = 41.7 +/− 22.0 (2SD) and FOM = 0.29) comprised 12 Se sites (occ > 0.3) and, after phase-and-build cycles, produced a preliminary 3D model of BT0996-C-His with 148 amino-acid residues, corresponding to 2 monomers in the asymmetric unit, which refined to R_work_ = 0.38 and R_free_ = 0.44 (overall model-map correlation = 0.678) using phenix.refine (Afonine et al., 2012).

#### His-BT0996-C with MPD

X-ray diffraction data from crystals of His-BT0996-C with MPD were collected at the BL-13 XALOC beamline of the ALBA Synchrotron (Barcelona, Spain) to a maximum resolution of 1.65 Å and using radiation of 0.976 Å wavelength. The diffraction data were indexed in *P*2_1_2_1_2_1_ space group with cell constants *a* = 34.98 Å, *b* = 45.94 Å, and *c* = 85.82 Å and processed using *autoPROC* software package (Vonrhein et al., 2011) from the beamline pipeline. Matthews Coefficient calculations suggested the presence of 1 copy of His-BT0996-C in the asymmetric unit, with V_M_ = 2.18 Å^3^Da^−1^ and an approximate solvent content of 44 %. The structure was solved by molecular replacement with *PhaserMR* (McCoy et al., 2007), using as search model one monomer of the preliminary 3D structure of SeMet BT0996-C-His produced by AutoSol. All subsequent refinement cycles were carried out in the *Phenix* platform (Adams et al., 2010), using program *phenix.refine* (Afonine et al., 2012), alternated with manual building and validation of the structure in *Coot* (Emsley and Cowtan, 2004). A *molprobity* check was run automatically by the program *phenix.refine* after each refinement cycle. The final His-BT0996-C structure has 131 amino-acid residues. All data collection and refinement statistics are summarised in Supplementary Table S5.

### Carbohydrate microarray analysis

The His-BT0996-C CBM was analysed in a microarray consisting of 20 polysaccharides derived from plants, of which seven of these are pectic. The pectin polysaccharides were polygalacturonic acid (PGA), with a backbone of α-1,4 Galacturonic acid (GalA); rhamnogalacturonan I, with a mixed-linked α-1,4-GalA and α-1,2 Rhamnose (Rha) backbone and galactan, with a β-1,4 galactose (Gal) backbone. All these pectic polysaccharides have ramifications with diverse monosaccharide composition. PGA and Galactan polysaccharide probes are from diverse sources. The Plant-related polysaccharide array covered a diversity of sequences used in previous works (Rudkin et al., 2018). Polysaccharide information of each probe and details of the microarray data and metadata are in the supplementary glycan microarray document (Table S9) in accordance with the Minimum Information Required for A Glycomics Experiment (MIRAGE) guidelines for reporting glycan microarray-based data (Liu et al., 2017). Polysaccharides were taken up in water, except for curdlan polysaccharide which was solubilised using mild alkaline solution (50 mM NaOH).

The microarrays were analysed with His-BT0996-C, following described protocols (Liu et al., 2012; Palma et al., 2015). In parallel, a CBM from family 35 (*Ct*CBM35, Cthe_2811) and four commercial monoclonal antibodies (anti-β1,4-Galactan LM5, anti-β1,4-Mannan, anti-β1,3-Glucan, and anti-β1,3/1,4-Glucan) of known specificity were used as control proteins in the microarray assays. These proteins are detailed in Supplementary Table S2. Nitrocellulose nonspecific binding sites were blocked using the blocking solution of 1 % BSA (Sigma-Aldrich, A8577) and 0.02 % Casein (Thermo Scientific, Waltham, MA, USA, 37583) diluted in respective assay buffer. The His-BT0996-C CBM and the monoclonal antibodies were analysed at a final concentration of 10 µg/mL, and the Cthe_2811 *Ct*CBM35 was analysed at a final concentration of 5 µg/mL. The CBMs were pre-complexed with mouse monoclonal anti-polyhistidine (Sigma-Aldrich, H1029) (Ab1) and biotinylated anti-mouse IgG antibodies (Sigma-Aldrich, B7264) (Ab2) at a ratio of 1:3:3 (by weight). The CBM-antibody complexes were prepared by preincubating Ab1 with Ab2 for 15 min at 20 °C, followed by the addition of the CBM and further incubation for 15 min. The binding was detected by Alexa Fluor-647-labeled streptavidin at 1 μg/ml (Molecular Probes, S21374) diluted in the blocking solution. Binding assays were conducted at room temperature.

All microarray slides were scanned with GenePix® 4300A fluorescence scanner, and the fluorescence was quantified using GenePix® Pro Software (both from Molecular Devices). Microarray data analysis was performed using dedicated software developed by Dr. Mark Stoll from the Glycosciences Laboratory (Imperial College London, UK) (Stoll and Feizi, 2009).

### Microscale thermophoresis experiments

Microscale thermophoresis experiments were carried out on a Monolith NT.115 (red/blue) instrument (NanoTemper Technologies, Munich, Germany). The purified CBM BT0996-C-His was labelled with Monolith NT™ Protein Labeling Kit RED-NHS, following the manufacturer's protocol. The experiments were performed with BT0996-C in HEPES pH 7.5, 100 mM NaCl, 5 mM CaCl_2_, 5 mM TCEP with Tween®20 0.05 % (v/v) at a final concentration of 66 nM. The oligosaccharides' dilution series was prepared in the buffer of the labelled BT0996-C (16 concentration points, ranging from 76.3 nM to 2.5 mM). BT0996-C was mixed with each ligand solution. The mixtures were incubated for 15 min at room temperature and then loaded into standard treated capillaries. The experiments were recorded at 22 °C (40 % Excitation Power and 40 % MST Power) using the MO.control software v1.4. The quenching of the initial fluorescence, instead of the thermophoresis, was used to calculate the binding dissociation constant of the interaction. An SDS-denaturation test was performed to confirm that the quenching resulted from the specific interaction of the galacturonic oligosaccharides with the BT0996-C protein. The replicates of independent measurements were analysed with M.O.Affinity Analysis Software v2.3.

## Author contributions

ALC, ASP and BAP designed and supervised the work. The experiments were conducted by FT, VGC, FL, DR and BAP. FT, VGC, BAP, ALC and ASP prepared and revised the manuscript.

## CRediT authorship contribution statement

**Filipa Trovão:** Investigation, Formal analysis, Visualization, Writing – original draft, Writing – review & editing. **Viviana G. Correia:** Investigation, Formal analysis, Writing – review & editing. **Frederico M. Lourenço:** Investigation. **Diana O. Ribeiro:** Investigation. **Ana Luísa Carvalho:** Conceptualization, Supervision, Writing – review & editing. **Angelina S. Palma:** Conceptualization, Supervision, Funding acquisition, Writing – review & editing. **Benedita A. Pinheiro:** Conceptualization, Supervision, Investigation, Formal analysis, Writing – original draft, Writing – review & editing.

## Declaration of Competing Interest

The authors declare that they have no known competing financial interests or personal relationships that could have appeared to influence the work reported in this paper.

## Data Availability

Data will be made available on request.
