# Supplementary material for: The structure of a Bacteroides thetaiotaomicron carbohydrate-binding module provides new insight into the recognition of complex pectic polysaccharides by the human microbiome
Source: J Struct Biol X. 2023 Jan 2;7:100084. doi: 10.1016/j.yjsbx.2022.100084 (PMC9843283; doi:10.1016/j.yjsbx.2022.100084)
Supplement: Supplementary data 1 [file mmc1.pdf]

Supplementary Material

The structure of a *Bacteroides thetaiotamicron* carbohydrate-binding module provides new insight into the recognition of complex pectic polysaccharides by the human microbiome

Filipa Trovão[1,2,§], Viviana G. Correia[1,2,§], Frederico Lourenço[1,2,•], Diana O. Ribeiro[1,2], Ana Luísa Carvalho[1,2], Angelina S. Palma[1,2,*] and Benedita A. Pinheiro[1,2,*]

1 UCIBIO – Applied Molecular Biosciences Unit, Department of Chemistry, NOVA School of Science and Technology, Universidade NOVA de Lisboa, 2829-516 Caparica, Portugal;
2 Associate Laboratory i4HB - Institute for Health and Bioeconomy, NOVA School of Science and Technology, Universidade NOVA de Lisboa, 2829-516 Caparica, Portugal

§ Authors contributed equally
• New Address: ITQB – Instituto de Tecnologia Química e Biológica António Xavier, Universidade NOVA de Lisboa, Av. da República, 2780-157 Oeiras, Portugal

* Corresponding authors:
Benedita A. Pinheiro, UCIBIO, Departamento de Química, Faculdade de Ciências e Tecnologia, Universidade NOVA de Lisboa, 2829-516 Caparica, Portugal; Tel: +351 212948300; E-mail: b.pinheiro@fct.unl.pt
Angelina S. Palma, UCIBIO, Departamento de Química, Faculdade de Ciências e Tecnologia, Universidade NOVA de Lisboa, 2829-516 Caparica, Portugal; Tel: +351 212948300; E-mail: ma.palma@fct.unl.pt;

List of Supplementary Material

**Figure S1**	Schematic representation of Rhamnogalacturonan-II (RG-II) primary structure.
**Figure S2**	Carbohydrate microarray analysis of Anti-(1-4)-β-D-mannan, of Anti-(1-4)-β-D-glucan and Anti-(1-3;1-4)-β-D-glucan to plant-related polysaccharides.
**Figure S3**	Details of the ribbon representation of the crystal structure of the BT0996-C CBM module. A. Coordination geometry of the structural calcium ion. B. MPD molecule in BT0996-C putative binding site.
**Table S1**	Polysaccharide samples included in the microarray, sorted by backbone type and main glycosidic linkage.
**Table S2**	Reported carbohydrate-binding specificity and source of selected Carbohydrate-Binding Module and Monoclonal Antibodies used as validation and control proteins in the microarray assays.
**Table S3**	Fluorescence binding intensities elicited with BT0996-C CBM and selected control proteins.
**Table S4**	Fluorescence binding intensities elicited with BT0996-C CBM to the Polygalacturonic Acid Polysaccharides at different ionic strengths.
**Table S5**	X-ray crystallography parameters and statistics.
**Table S6**	List of Monosaccharides used in co-crystallisation experiments.
**Table S7**	PDBeFold Top 10 BT0996-C Homologue Structures, ordered by Q-score. Q-score represents the quality function of Cα-alignment, maximised by the SSM (Secondary Structure Matching) alignment algorithm. Q-score considers the alignment length (Nalign) and RMSD.
**Table S8**	Oligonucleotides used for cloning and re-cloning of BT0996-C.
**Table S9**	Glycan microarray document based on MIRAGE Glycan Microarray Guidelines
**Supplementary References**	

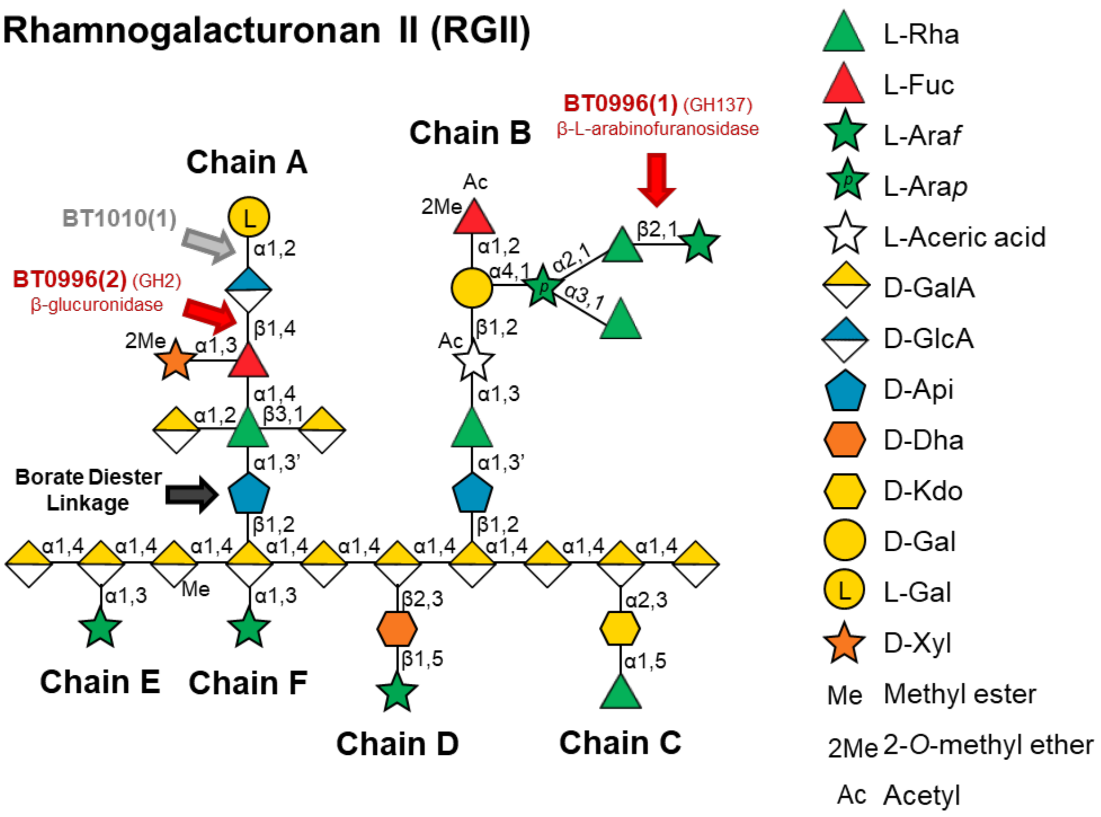

Figure S1. Schematic representation of Rhamnogalacturonan-II (RG-II) primary structure. The monosaccharide sequence of RG-II was represented using the updated symbol nomenclature for glycans (SNFG) (Neelamegham et al. 2019). BT1010 and BT0996 enzymes' sites of action are indicated by grey and red arrows, respectively. Values in parentheses indicate the predicted order of action (Ndeh et al. 2017). A black arrow indicates the location of the borate diester linkage responsible for RG-II dimerization.

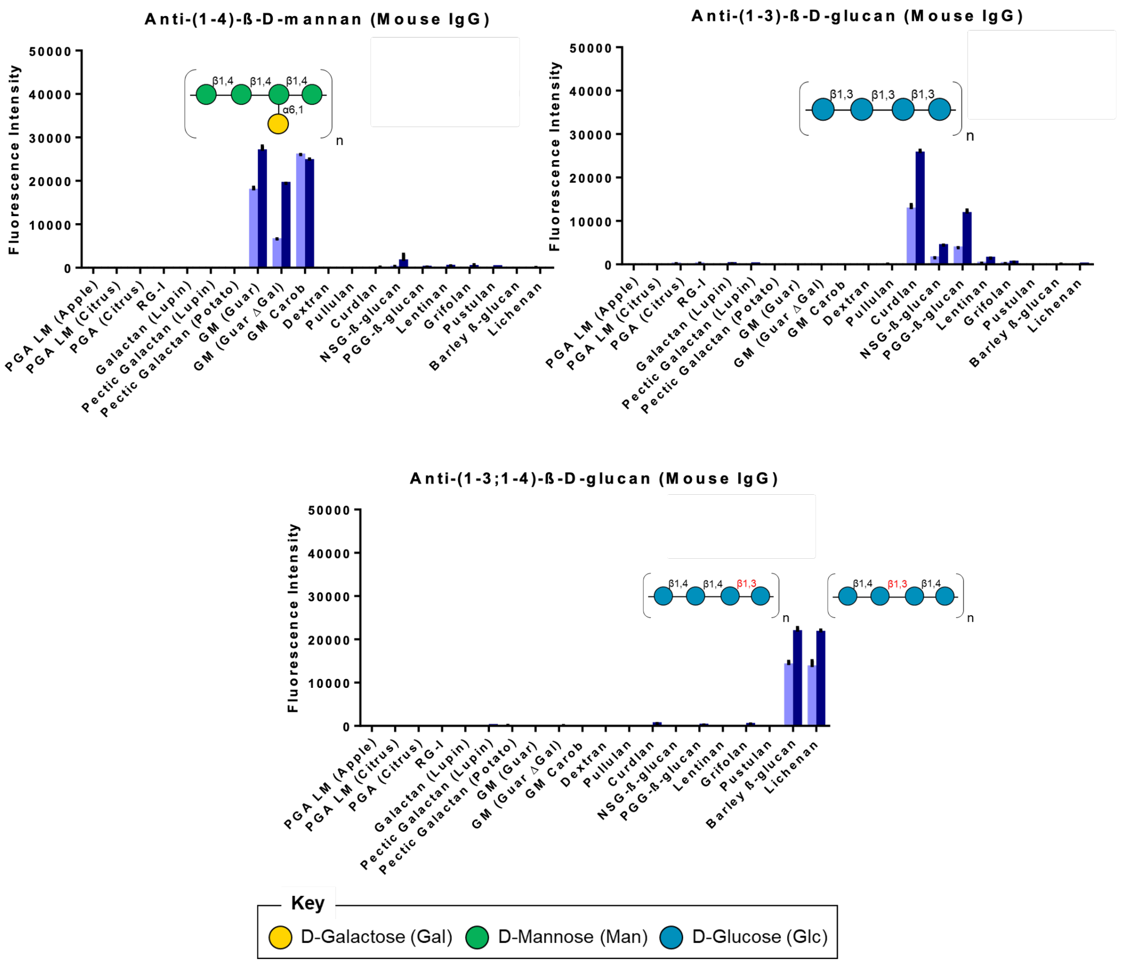

Figure S2. Carbohydrate microarray analysis of Anti-(1-4)-β-D-mannan, of Anti-(1-4)-β-D-glucan and Anti-(1-3;1-4)-β-D-glucan to plant-related polysaccharides. The binding scores are depicted as fluorescence intensities elicited with 30 and 150 pg (by weight) polysaccharide per spot (light blue and dark blue, respectively). The major oligosaccharide sequence domains present in the bound polysaccharides are represented using the updated symbol nomenclature for glycans (SNFG) (Neelamegham et al. 2019). PGA, Polygalacturonic Acid; LM, Low Methoxyl; RG-I, Rhamnogalacturonan I; GM, Galactomannan; NSG, Neutral Soluble Glucan; PGG, Poly-(1,6)-D-glucopyranosyl-(1,3)-D-glucopyranose.

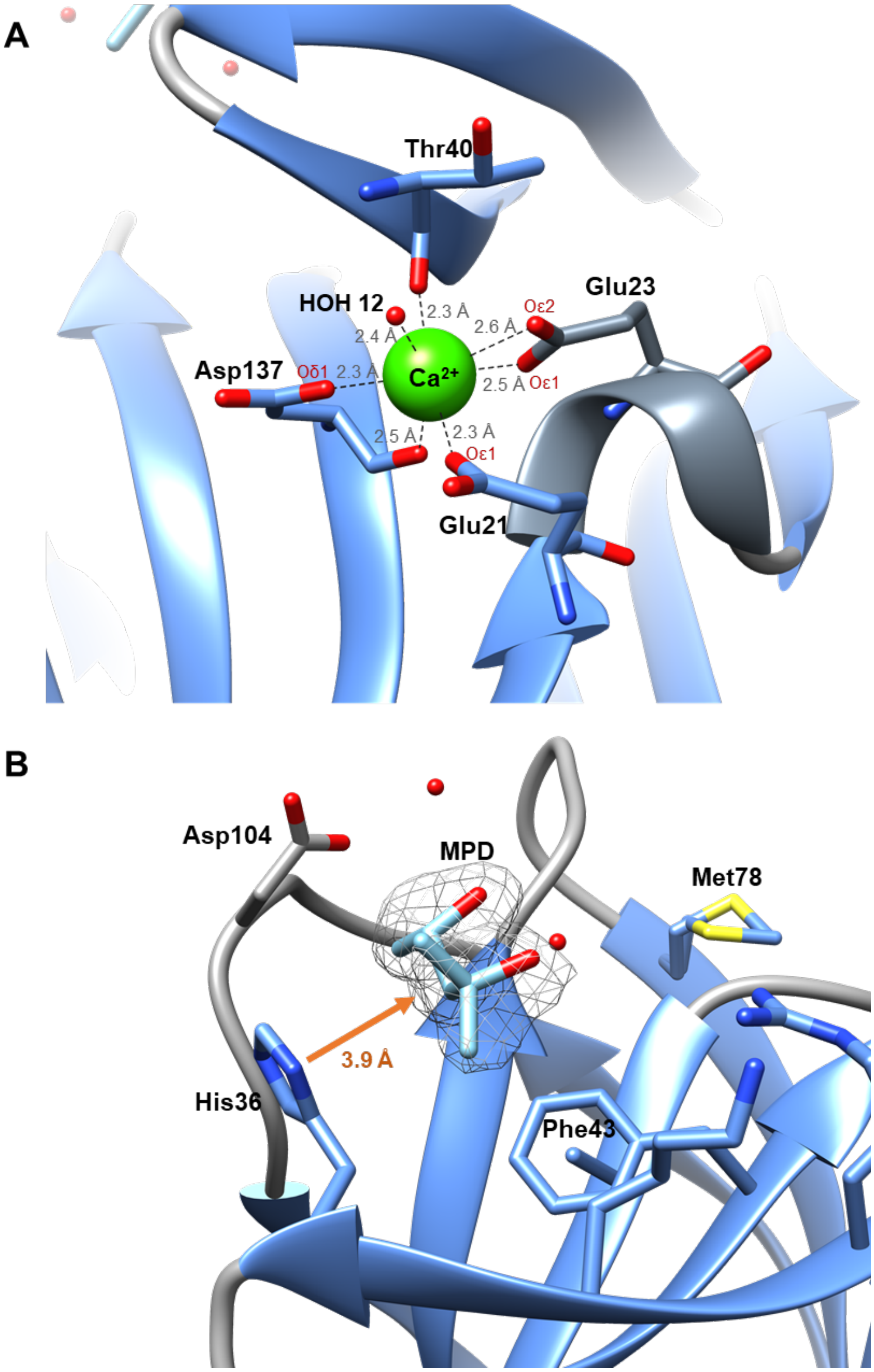

Figure S3. Details of the crystal structure of BT0996-C CBM module. A. Coordination geometry of the structural calcium ion. B. MPD molecule in BT0996-C putative binding site. Amino acid residues, water molecules and the MPD molecule are shown in stick representation. The atomic distances of the coordinating atoms to the calcium ion and of the MPD to the His36 residue are displayed. The calcium (II) ion is represented by a green sphere. The final $2mF_o$-DF_c electron density map surrounding the MPD is contoured at 1 σ. The structural representations were performed with UCSF Chimera (Pettersen et al., 2004).

Table S1. Polysaccharide samples included in the microarray, sorted by backbone type and main glycosidic linkage.

ID[a]	Polysaccharide Probe[b]	Biological Source & Supplier[c]	Predominant oligosaccharide sequence/Monosaccharide composition
1	Polygalacturonic Acid Low Methoxyl (PGA LM) (Apple)	Apple pectins; Low methylated; Elicityl (GAT100)	α1,4-GalA backbone with α1,2-Rha and other side chains
2	Polygalacturonic Acid Low Methoxyl (PGA LM) (Citrus)	Citrus pectins; Low methylated; Elicityl (GAT102)	α1,4-GalA backbone with α1,2-Rha and other side chains; Ara (0.21%), Rha (0.42%), Fuc (0.01%), Xyl (1.5%), Gal (3.67%), Glc (0.94%), UA (92.6%)
3	Polygalacturonic Acid (PGA) (Citrus)	Citrus; Megazyme (P-PGACT)	α1,4-GalA backbone with possible ramifications; Ara (0.81%), Rha (0.47%), Fuc (0.01%), Xyl (0.09%), Gal (4.51%), Glc (3.03%), UA (88.6%)
4	Rhamnogalacturonan (Soybean)	Soybean; Megazyme (P-RHAGN)	Mixed-linked α1,4-GalA and α1,2-Rha backbone with ramifications; GalA (51%), Rha(13%), Fuc (21%), Ara (7%), Xyl (28%), Gal (25%), other neutral sugars (3%)
5	Galactan (Lupin)	Lupin; Arabinofuranosidase treated pectic galactan; Megazyme (P-GALLU)	β1,4-Gal backbone with possible ramifications; Neutral sugars: Gal (82%), Ara (5.8%), Rha (5.1%), Xyl (1.4%), other (5.7%); and GalA (14.6%)
6	Pectic Galactan (Lupin)	Lupin; Megazyme (P-PGALU)	β1,4-Gal backbone with possible ramifications; Gal (77%), Ara (14%), Rha (3%), Xyl (0.6%), GalUA (5.4%)
7	Pectic Galactan (Potato)	Potato; Megazyme (P-PGAPT)	β1,4-Gal backbone with possible ramifications; Gal (78%), Ara (9%), Rha (4%), GalUA (9%)
8	Galactomannan (Guar)	Guar; Megazyme (P-GGMMV)	β1,4-Man backbone with α1,6-Gal ramifications; Gal (38%), Man (62%)
9	Galactomannan (Guar, ΔGal)	Guar; Gal depleted; Megazyme (P-GGM21)	β1,4-Man backbone with α1,6-Gal ramifications; Gal (21%), Man (79%)
10	Galactomannan (Carob)	Carob; Megazyme (P-GALML)	β1,4-Man backbone with α1,6-Gal ramifications; Gal (24%), Man (76%)
11	Dextran	*Leuconostoc mesenteroides*; Sigma-Aldrich (D4876)	α1,6-Glc
12	Pullulan	*Aureobasidium pullulans*; Megazyme (P-PULLN)	Mixed-linked α1,4/1,6-Glc (α1,6-linked maltotriosyl repeats)
13	Curdlan	*Agrobacterium* sp., strain ATCC31749	β1,3-Glc
14	NSG-β-glucan (Neutral soluble glucan)	*Saccharomyces cerevisiae;* Biothera	β1,3-Glc backbone with occasional monoglucosyl β1,6-Glc branches
15	PGG-β-glucan (Poly-(1,6)-D-glucopyranosyl-(1,3)-D-glucopyranose)	*Saccharomyces cerevisiae;* Biothera	β1,3-Glc backbone with occasional monoglucosyl β1,6-Glc branches
16	Lentinan	*Lentinus edodes*	β1,3-Glc backbone with occasional monoglucosyl β1,6-Glc branches
17	Grifolan	*Grifola frondosa*	β1,3-Glc backbone with highly ramified oligomeric β1,6-Glc branches
18	Pustulan	*Umbilicaria papullosa*; Elicityl (GLU900)	β1,6-Glc
19	Barley β-glucan	Barley flour; Megazyme (P-BGBL)	Mixed-linked β1,3/1,4-Glc; 1:3-4 linkage ratio; contains Ara (2%), Xyl (0.2%)
20	Lichenan	Icelandic moss; Megazyme (P-LICHN)	Mixed-linked β1,3/1,4-Glc; 1:2 linkage ratio

[a] ID number corresponding to the probe position in the binding chart.

[b] Probes are grouped according to predominant oligosaccharide sequence and glycosidic linkage or monosaccharide composition.
[c] Sources are indicated for each carbohydrate sample; if commercial, the product code is in brackets.

Table S2. Reported carbohydrate-binding specificity and source of selected Carbohydrate-Binding Module and Monoclonal Antibodies used as validation and control proteins in the microarray assays.

Protein	Source	Reported glycan recognition	Reference[a]
**Family 35 carbohydrate-binding module**			
***Ct*CBM35 (Cthe_2811)**	*Clostridium thermocellum;* Recombinant (His-tag)	Galactomannans (Man-Gal-Man moiety) and Mannotriose	(Ghosh et al. 2014)
**Monoclonal Antibodies**			
**LM5 Anti-β1,4-Galactan**	Rat IgG; Plant probes (LM5-050)	β1,4-galactosyl residues found in the galactan components of certain pectic polymers; Linear tetrasaccharide in β1-4-D-galactan	(Jones, Seymour, and Knox 1997)
**400-4 Anti-β1,4-Mannan**	Mouse IgG; Biosupplies (400-4)	Linear β1,4-Man oligosaccharides segments in β1,4-D-mannans and -galactomannans	(Pettolino et al. 2001)
**400-2 Anti-β1,3-Glucan**	Mouse IgG; Biosupplies (400-2)	Linear β1,3-Glc oligosaccharide segments in β1,3-D-glucans	(P. J. Meikle et al. 1991)
**400-3 Anti-β1,3/1,4-Glucan**	Mouse IgG; Biosupplies (400-3)	Linear β1,3/1,4-Glc oligosaccharide segments in mixed-linkage β1,3/1,4-D-glucans	(Peter J. Meikle et al. 1994)

[a] Main carbohydrate-binding supported by a particular study.

Table S3. Fluorescence binding intensities elicited with BT0996-C CBM and control proteins.

ID[a]	Probe[b]	Score±Err[c]											
		BT0996-C		*Ct*CBM35 (Cthe_2811)		Anti-β1,4-Galactan LM5		Anti-β1,4-Mannan		Anti-β1,3-Glucan		Anti-β1,3/1,4-Glucan	
		Low Level	High Level	Low Level	High Level	Low Level	High Level	Low Level	High Level	Low Level	High Level	Low Level	High Level
1	PGA LM (Apple)	11710.5 ±299.5	28874.5 ±1478.5	-	-	10733 ±137	23364.5 ±582.5	-	-	-	-	-	-
2	PGA LM (Citrus)	3944 ±212	17452 ±620	-	-	6561.5 ±224.5	21305 ±196	-	-	-	-	-	-
3	PGA (Citrus)	4965.5 ±613.5	19056 ±127	-	-	9879.5 ±26.5	26821.5 ±648.5	-	-	-	-	-	-
4	RG-I	-	-	-	-	10970 ±81	32192 ±389	-	-	-	-	-	-
5	Galactan (Lupin)	-	-	-	-	34639 ±182	39469 ±1865	-	-	-	-	-	-
6	Pectic Galactan (Lupin)	-	-	-	-	40171 ±1259	30087.5 ±1578.5	-	-	-	-	-	-
7	Pectic Galactan (Potato)	-	2429.5 ±2.5	-	-	38019 ±978	36148 ±454	-	-	-	-	-	-
8	GM (Guar)	-	-	11568.5 ±242.5	22324.5 ±561.5	-	-	17821 ±713	26890.5 ±1130.5	-	-	-	-
9	GM (Guar ∆Gal)	-	-	3455.5 ±483.5	12322 ±656	-	-	6451 ±174	19394 ±25	-	-	-	-
10	GM Carob	-	-	20998.5 ±20.5	40492 ±404	-	-	25900 ±177	24659 ±381	-	-	-	-
11	Dextran	-	-	-	-	-	-	-	-	-	-	-	-
12	Pullulan	-	-	-	-	-	-	-	-	-	-	-	-
13	Curdlan	-	-	-	-	-	-	-	-	12721.5 ±1058.5	25620.5 ±669.5	-	-
14	NSG-β-glucan	-	-	-	-	-	-	-	-	1464 ±45	4311 ±108	-	-
15	PGG-β-glucan	-	-	-	-	-	-	-	-	3758 ±96	11679 ±824	-	-
16	Lentinan	-	-	-	-	-	-	-	-	-	1401.5 ±25.5	-	-
17	Grifolan	-	-	-	-	-	-	-	-	-	-	-	-

18	Pustulan	-	-	-	-	-	-	-	-	-	-	-	-
19	Barley β-glucan	-	-	-	-	-	-	-	-	-	-	14068 ±814	21768 ±958
20	Lichenan	-	-	-	-	-	-	-	-	-	-	13593 ±1485	21611.5 ±550.5

[a] ID number corresponding to the probe position in the binding chart.
[b] Carbohydrate probes are all polysaccharides, organised by predominant oligosaccharide composition and glycosidic linkage or monosaccharide composition.
[c] Fluorescence binding signals are shown as means of duplicate spots at 30pg/spot and 150pg/spot (by weight) of each carbohydrate probe with respective error (Err).
(-) Binding signal below background (<1000).

Table S4. Fluorescence binding intensities elicited with BT0996-C CBM to the Polygalacturonic Acid Polysaccharides at different ionic strengths.

Polysaccharide sample	Score±Err[c]					
	HEPES Buffer pH 7.5			Tris-HCl Buffer pH 8.5		
	0 mM NaCl	150 mM NaCl	300 mM NaCl	0 mM NaCl	150 mM NaCl	300 mM NaCl
PGA LM (Apple)	23338.5 ±155.5	28874.5 ±1478.5	-	24751.5 ±1240.5	-	-
PGA LM (Citrus)	14056.5 ±21.5	17452 ±620	-	15220.5 ±281.5	-	-
PGA (Citrus)	15325 ±508	19056 ±127	-	11064 ±350	-	-

[a] ID number corresponding to the probe position in the binding chart.
[c] Fluorescence binding signals are shown as means of duplicate spots at 150pg (by weight)/spot of each carbohydrate probe with respective error (Err).
(-) Binding signal below background (<1000).

Table S5. X-ray crystallography parameters and validation statistics for data collection, 3D structure solution and refinement. Values for the high-resolution shell are shown in parentheses.

	BT0996-C SeMet	*BT0996-C*
	Data collection and processing	
X-ray source	ESRF, ID30B	ALBA, XALOC BL-13
Wavelength (Å)	0.97937	0.976
Space group	$P4_1\ 2\ 2$	$P2_1\ 2_1\ 2_1$
Unit-cell parameters (Å, °)	*a* = *b* = 49.14, *c* = 282.89, α = β = γ = 90	*a* = 34.98, *b* = 45.94, *c* = 85.82, α = β = γ = 90
Resolution range (Å)	48.92 - 2.34 (2.42 - 2.34)	32.39 - 1.65 (1.71 - 1.65)
Matthews coefficient, V_M(Å^3/Da)	2.84	2.18
Solvent content (%)	57.0	43.5
Protein molecules per asymmetric unit	2	1
Average mosaicity (°)	0.45	0.14
Mean *I/σ(I)*	7.7 (2.6)	15.4 (2.4)
Wilson B-factor (Å^2)	37.8	20.2
R_{merge}†)	0.118 (0.561)	0.023 (0.23)
$R_{p.i.m.}$$^+$	0.070 (0.398)	0.023 (0.23)
Half-dataset correlation CC1/2	0.987 (0.671)	0.99 (0.92)
Multiplicity	5.9 (4.8)	2.0 (2.0)
Anomalous multiplicity	3.2 (2.6)	-
Total reflections	90097 (7006)	34395 (3361)
Unique reflections	15165 (1460)	17236 (1683)
Completeness (%)	98.2 (99.1)	99.71 (99.64)
Anomalous completeness (%)	97.1 (96.6)	-
BAYES-CC	41.7 +/- 22.0	
FOM for 12 Se sites	0.29	-
	Refinement statistics	
Protein atoms	1547	1069
Ligand atoms	-	15
Solvent molecules	-	129
R_{work}‡	0.38	0.174 (0.281)
R_{free}§	0.44	0.225 (0.327)
Overall model-map correlation	0.678	0.897
R.m.s.d. bond lengths (Å)	-	0.007
R.m.s.d. bond angles (º)	-	0.88
Average B-factor (Å^2)	-	27.55
Protein	-	26.48
MPD	-	42.70
Ca^{2+}	-	18.28
Cl^-	-	55.03
Water molecules (129)	-	34.42
Ramachandran plot		
Residues in favoured regions (%)	-	97.7
Residues in allowed regions (%)	-	2.3
Residues outliers (%)	-	0.0
PDB accession code	-	7ZVO

† $R_{merge} = \frac{\sum_{hkl} \sum_{i=1}^{n} |I_i(hkl) - \underline{I}(hkl)|}{\sum_{hkl} \sum_{i=1}^{n} I_i(hkl)}$, where I is the observed intensity, and $\underline{I}$ is the statistically-weighted average intensity of multiple observations. $^+$ $R_{p.i.m.} = \frac{\sum_{hkl} \sqrt{\frac{1}{(n-1)}} \sum_{i=1}^{n} |I_i(hkl) - \underline{I}(hkl)|}{\sum_{hkl} \sum_{i=1}^{n} I_i(hkl)}$, a redundancy-independent version of R_{merge}. ‡ $R_{work} = \frac{\sum_{hkl} ||F_{obs}(hkl)| - |F_{calc}(hkl)||}{\sum_{hkl} |F_{obs}(hkl)|}$, where $|F_{calc}|$ and $|F_{obs}|$ are the calculated and observed structure factor amplitudes, respectively. § R_{free} is calculated for a randomly chosen 5% of the reflections.

Table S6. Monosaccharides used in co-crystallisation experiments.

Monosaccharide	MW (g/mol)	Company
D-fucose	164.16	Merck
D-galacturonic monohydrate	212.15	Sigma-Aldrich
D-glucuronic acid	194.14	Sigma-Aldrich
L-arabinose	150.13	Sigma-Aldrich
L-galactose	180.16	Sigma-Aldrich
L-rhamnose	182.17	Merck

Table S7. PDBeFold Top 10 BT0996-C Homologue Structures, ordered by Q-score. Q-score represents the quality function of Cα-alignment, maximised by the SSM (Secondary Structure Matching) alignment algorithm. Q-score considers the alignment length (N_{align}) and RMSD.

##	PDB	Q-score	RMSD	N_{align}	% Seq Ident	Title
1	**2vzq**	0.59	1.75	115	21	Chi-**CBM35** of the exo-β-D-glucosaminidase from *A. orientalis*
2	**2w47**	0.55	1.77	115	22	Rhe-**CBM35** of rhamnogalacturonan acetyl esterase from *C. thermocellum*
3	**5fui**	0.53	2.03	113	17	ZgLamC-**CBM6** of the laminarinase from *Zobellia galactanivorans*
4	**4qb6**	0.51	1.83	109	20	Xyn30D-**CBM35** of the glucuronoxylanase Xyn30D from *Paenibacillus barcinonensis*
5	**2cdp**	0.50	2.36	117	14	Aga16B-**CBM6**-2 of the β-agarase from *Saccharophagus degradans 2-40*
6	**2wz8**	0.49	1.99	112	17	**CBM35** binding D-galactose from *C. thermocellum*
7	**1ux7**	0.48	2.28	110	11	**CBM36** domain of the *Paenibacillus polymyxa* xylanase
8	**2w87**	0.48	1.97	113	17	Xyl-**CBM35** of the xylanase CjXyn10B from *Cellvibrio japonicus*
9	**1uxz**	0.48	2.32	115	11	*Cm***CBM6**-2 of endoglucanase 5A from *Cellvibrio mixtu*
10	**2w3j**	0.47	1.98	111	17	Pel-**CBM35** a PL10 pectate lyase (Pel10) from an environmental isolate

Table S8. Oligonucleotides used for re-cloning of BT0996-C.

Construct	Oligonucleotide Sequence (5'→3') [a]	Vector
BT0996-C-**His**	(F) CAC **CCATGG** GCTATGAAGCTGAAACGGCAAC<br>(R) CTC **CTCGAG** AATATCCAAGGCATCAAAAGCC	pET28a vector; C-terminal 6xHis tag;

[a] Forward primer (F) and Reverse primer (R); LIC vector complementary sequences are underlined.

Table S9. Glycan microarray document based on MIRAGE Glycan Microarray Guidelines (doi:10.3762/mirage.3) (Liu et al. 2017).

Classification	Guidelines
**1. Sample: Glycan Binding Sample**	
Description of Sample	Sample names: **BT0996-C**, CBM from commensal bacterium *Bacteroides thetaiotaomicron VPI-5482*<br>Origin: Recombinant<br>Method of preparation: Please see the *Methods* section in the main text.
Sample modifications	Not relevant.
Assay protocol	Please see Methods section in the main text.
**2. Glycan Library**	
Glycan description for defined glycans	The "Plant-related Polysaccharide Microarray" featuring 20 polysaccharide probes derived from plants and pectins. The polysaccharide samples, monosaccharide composition/predominant oligosaccharide sequences and sources are in Supplementary Table S4. Some of the polysaccharides have been described previously (Palma et al. 2015).
Glycan description for undefined glycans	Not relevant.
Glycan modifications	Polysaccharides were not modified.
**2. Printing Surface; e.g., Microarray Slide**	
Description of surface	Nitrocellulose-coated glass microarray slides.
Manufacturer	16-pad UniSart® 3D Microarray Slide from Sartorius (Goettingen, Germany).
Custom preparation of surface	Not relevant.
Non-covalent Immobilisation	Polysaccharides were immobilised non-covalently without any formulation.
**4. Arrayer (Printer)**	
Description of Arrayer	Nano-Plotter 2.1 (GeSiM, Radeberg, Germany).
Dispensing mechanism	Non-contact liquid delivery with four dispensing tips.
Glycan deposition	Approximately 0.33 nL was printed per spot.<br>Polysaccharides were printed at two levels, 30 and 150 pg (by weight) per spot, in duplicate.
Printing conditions	The printing solutions were aqueous-based. Printing was performed at ambient temperature and relative humidity of 58%.<br>The printing solutions contained polysaccharides at 0.1 and 0.3 mg dry weight/mL for the 0.03 and 0.1 ng per spot levels, respectively.<br>The printing solutions also contained Cy3 NHS ester (GE Healthcare) at 20 ng/mL (26 fmol/μL) as a marker to monitor the printing process.
**5. Glycan Microarray with "Map"**	
Array layout	The arrayed slides contained 16 identical pads (subarrays). Each pad was set up for printing 64 probes maximum, each at 2 levels in duplicate (four spots for one probe in a row); 256 spots (16x16) in total for 64 probes. When not completely printed, the remaining space in each pad was treated as "blank" to allow quantification using the same grid; "blank probes" were excluded from final data presentation.

Glycan identification and quality control	Quality control was performed with the sequence-specific monoclonal antibodies: Anti-β1,4-Galactan LM5, Anti-β1,4-Mannan, Anti-β1,3-Glucan, Anti-β1,3/1,4-Glucan. The microarray was also analysed with a carbohydrate-binding module from family 35, the CtCBM35 (Cthe_2811) from *Clostridium thermocellum* that recognises mannan and galactomannan polysaccharides (Ghosh et al. 2014).<br>All proteins and their reported carbohydrate binding are listed in Supplementary Table S5. The predicted binding was observed for all the proteins.
**6. Detector and Data Processing**	
Scanning hardware	GenePix 4300A (Molecular Devices, UK)
Scanner settings	Scanning resolution: 10 μm / pixel;<br>Laser channel: Red (scan wavelength 635nm);<br>PMT Voltages: 350<br>Scan powers: 5%, 10%, 30%, 70% or 90% to achieve maximum signal without spot saturation.
Image analysis software	GenePix® Pro 7 (Molecular Devices, UK)
Data processing	The gpr file was entered into an in-house microarray database using software (designed by Mark Stoll, http://www.beilstein-institut.de/en/publications/proceedings/glyco-2009) for data processing. No particular normalisation method or statistical analysis was used.
**7. Glycan Microarray Data Presentation**	
Data presentation	The microarrays binding results are depicted in Figure 4 and S3, and in Supplementary Table S6 and S7.
**8. Interpretation and Conclusion from Microarray Data**	
Data interpretation	No software or algorithms were used to interpret processed data.
Conclusions	BT0996-C showed exclusive binding with the pectin samples polygalacturonate (PGA) and galacturonates (PGA LM) from apple and citrus that contain the anionic and acidic α1,4-galacturonic chain.

Supplementary References

Du, Yuguo et al. 2004. "Synthesis and Antitumor Activities of Glucan Derivatives." *Tetrahedron* 60(30): 6345–51.

Ghosh, A. et al. 2014. "Structure and Functional Investigation of Ligand Binding by a Family 35 Carbohydrate Binding Module (CtCBM35) of β-Mannanase of Family 26 Glycoside Hydrolase from Clostridium Thermocellum." *Biochemistry (Moscow)* 79(7): 672–86.

Haworth, W. N., E. L. Hirst, and F. A. Isherwood. 1937. "Polysaccharides. Part XXIV. Yeast Mannan." *Journal of the Chemical Society (Resumed)* 160: 784–91.

Hong, Feng et al. 2003. "β-Glucan Functions as an Adjuvant for Monoclonal Antibody Immunotherapy by Recruiting Tumoricidal Granulocytes as Killer Cells." *Cancer Research* 63(24): 9023–31.

Jamas, S, D D Easson, G R Ostroff, and A B Onderdonk. 1991. "PGG-Glucans: A Novel Class of Macrophage-Activating Immunomodulators" eds. Richard L. Dunn and Raphael M. Ottenbrite. *Polymeric Drugs and Drug Delivery Systems, Vol. 469*: 44–51.

Jones, L., G. B. Seymour, and J. P. Knox. 1997. "Localisation of Pectic Galactan in Tomato Cell Walls Using a Monoclonal Antibody Specific to (1→4)-β-D-Galactan." Plant Physiology 113(4): 1405–12.

De la Cruz, J., J. A. Pintor-Toro, T. Benitez, and A. Llobell. 1995. "Purification and Characterisation of an Endo-β-1,6-Glucanase from Trichoderma Harzianum That Is Related to Its Mycoparasitism." *Journal of Bacteriology* 177(7): 1864–71.

Liu, Yan et al. 2017. "The Minimum Information Required for a Glycomics Experiment (MIRAGE) Project: Improving the Standards for Reporting Glycan Microarray-Based Data." *Glycobiology* 27(4): 280–84.

Mccleary, Barry V., and Norman K. Matheson. 1986. "Enzymic Analysis of Polysaccharide Structure" eds. R. Stuart Tipson and Derek Horton. *Advances in Carbohydrate Chemistry and Biochemistry* 44(C): 147–276.

Meikle, P. J. et al. 1991. "The Location of (1→3)-β-Glucans in the Walls of Pollen Tubes of Nicotiana Alata Using a (1→3)-β-Glucan-Specific Monoclonal Antibody." Planta 185(1): 1–8.

Meikle, Peter J. et al. 1994. "A (1→3,1→4)-β-glucan-specific Monoclonal Antibody and Its Use in the Quantitation and Immunocytochemical Location of (1→3,1→4)-β-glucans." The Plant Journal 5(1): 1–9.

Ndeh, Didier et al. 2017. "Complex Pectin Metabolism by Gut Bacteria Reveals Novel Catalytic Functions." *Nature* 544(7648): 65–70. http://dx.doi.org/10.1038/nature21725.

Neelamegham, Sriram et al. 2019. “Updates to the Symbol Nomenclature for Glycans Guidelines.” *Glycobiology* 29(9): 620–24.

Palma, Angelina S. et al. 2015. “Unravelling Glucan Recognition Systems by Glycome Microarrays Using the Designer Approach and Mass Spectrometry.” *Molecular & Cellular Proteomics* 14(4): 974–88.

Pettersen EF, et al. 2021 UCSF ChimeraX: Structure visualization for researchers, educators, and developers. Pettersen EF, Goddard TD, Huang CC, Meng EC, Couch GS, Croll TI, Morris JH, Ferrin TE. *Protein Sci.* 2021 Jan;30(1):70-82.

Pettolino, Filomena A. et al. 2001. “A (1→4)-β-Mannan-Specific Monoclonal Antibody and Its Use in the Immunocytochemical Location of Galactomannans.” Planta 214(2): 235–42.

Rudkin, Fiona M. et al. 2018. “Single Human B Cell-Derived Monoclonal Anti-Candida Antibodies Enhance Phagocytosis and Protect against Disseminated Candidiasis.” *Nature Communications* 9(1): 5288.

Wang, Xiaohua, Xiaojuan Xu, and Lina Zhang. 2008. “Thermally Induced Conformation Transition of Triple-Helical Lentinan in NaCl Aqueous Solution.” *The Journal of Physical Chemistry B* 112(33): 10343–51.

Yoo, Dong-Hyung et al. 2007. “Improved Quantitative Analysis of Oligosaccharides from Lichenase-Hydrolyzed Water-Soluble Barley β-Glucans by High-Performance Anion-Exchange Chromatography.” *Journal of Agricultural and Food Chemistry* 55(5): 1656–62.

Zhang, Hong-Tao et al. 2012. “Improved Curdlan Fermentation Process Based on Optimisation of Dissolved Oxygen Combined with PH Control and Metabolic Characterization of Agrobacterium Sp. ATCC 31749.” *Applied Microbiology and Biotechnology* 93(1): 367–79.
